# A Novel Homozygous Mutation of AIRE Gene in a Patient With Autoimmune Polyglandular Syndrome Type I

**DOI:** 10.7759/cureus.35374

**Published:** 2023-02-23

**Authors:** Camila M Tautiva-Rojas, Roberto Bogarin-Solano, Carlos Santamaría-Quesada, Mariana Pacheco-Muñoz

**Affiliations:** 1 Pediatrics, Hospital Nacional de Niños Dr. Carlos Saenz Herrera, San José, CRI; 2 Endocrinology, Hospital Nacional de Niños Dr. Carlos Saenz Herrera, San Jose, CRI; 3 Molecular Biology, Hospital Nacional de Niños Dr. Carlos Saenz Herrera, San José, CRI; 4 Neonatology, San Juan de Dios Hospital, San José, CRI

**Keywords:** european society for peadiatric endocrinology, addison's disease, chronic mucocutaneous candidiasis, aire mutation, severe hypocalcemia, mucocutaneous candidiasis, endocrine, autoimmune, gene, aire

## Abstract

Autoimmune polyglandular syndrome type I (APS1) shows common features such as mucocutaneous candidiasis, hypoparathyroidism, and hypoadrenalism. The clinical manifestations and their onset are highly variable. Besides endocrine abnormalities, patients can present with dental problems, keratoconjunctivitis, fever, rash, chronic diarrhea, and autoimmune hepatitis. We discuss the case of a 5-year-old female who presented initially with a new-onset seizure due to severe hypocalcemia and was diagnosed with primary hypoparathyroidism. Because she also had a history of chronic mucocutaneous candidiasis, chronic diarrhea, and the presence of autoantibodies tested positive, the diagnosis of APS1 was suspected. Genetic testing detected a novel pathogenic homozygous *AIRE* mutation, which confirmed the diagnosis. She began multidisciplinary treatment with antifungals, calcium supplements, and parathyroid hormone analogs.

## Introduction

Autoimmune polyendocrinopathy candidiasis ectodermal dysplasia also known as autoimmune polyglandular syndrome type I (APS1) is a rare recessive inherited disease caused by mutations in the gene *AIRE*, an autoimmune regulator. It is associated with endocrine and non-endocrine autoimmune diseases [1,2] and it's characterized by the triad of chronic mucocutaneous candidiasis, hypoparathyroidism, and Addison's disease [1]. It occurs at an approximate frequency of one per 90,000-200,000 people and it is more prevalent in genetically isolated groups of the population [2]. The diagnosis is often challenging and delayed given its various clinical and subtle manifestations. However, it can present clinically in children as young as three to five years old [3] and the peak of manifestations is usually at the age of 12 [2]. A slight female predominance has been reported, but both genders can be affected [1,2]. More than a hundred mutations of this gene have been described and associated with disease [3]. The course of the disease is very variable and differs in the number of affected systems and organs in each patient [2,3].

Here, we present a five-year-old female who presented with symptomatic hypocalcemia and a history of mucocutaneous candidiasis and chronic diarrhea. Genetic testing confirmed she had a homozygous mutation of the *AIRE* gene with pathologic significance that had not been previously described. 

This article was previously presented as a poster at the 60th European Society for Paediatric Endocrinology annual meeting in September 2022 at New Rome Congress Center, Rome, Italy. 

## Case presentation

**Figure 1:**
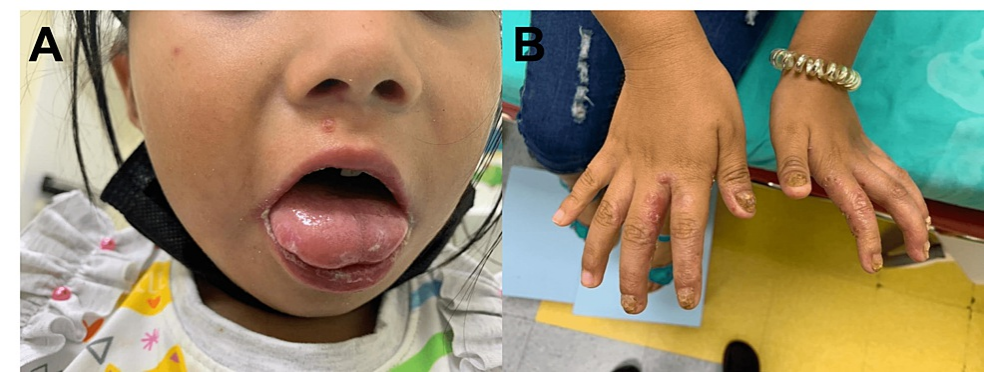
Chronic mucocutaneous candidiasis. (A) Candida lesions present as white plaques on the buccal mucosa and tongue and angular cheilitis; (B) Chronic changes due to fungal infection can be seen in fingers with red, pustular, thickened and crusted plaques; as well as in fingernails with both onychomycosis and paronychia.

A 5-year-old female child with a history of consanguinity presented to the emergency room with a new-onset tonic-clonic seizure without fever or other symptoms, no neurologic deficits during evaluation, and no prior history of epilepsy. She was admitted to the hospital for a workup, where she was found to have an electrolytic imbalance composed of severe hypocalcemia, hypomagnesemia, and hyperphosphatemia. She was found to be underweight with a BMI of 11.7kg/m^2^ (below the third percentile). It was also noted that since the age of two, she had several consults due to mucocutaneous candidiasis that didn’t improve with antifungal treatment (Figure 1), as well as frequent diarrhea episodes and dental problems, mostly cavities. 

The initial laboratory studies revealed a total calcium of 6.9 mg/dl (normal range: 8.8-10.8 mg/dl) and ionic calcium of 3 (normal range: 4.6-5.08 mg/dl). Her serum phosphate levels were elevated at 11.4 mg/dl (normal range: 3.2-5.8 mg/dl) and her parathormone (PTH) was extremely low at < 1.20 pg/ml (normal range: 1-13 pg/ml). Other biochemical and hormone findings are shown in Table 1. She was diagnosed with primary hypoparathyroidism and began treatment with intravenous calcium gluconate and a parathormone analog.

**Table 1:** Initial laboratory findings PTH: adrenocorticotropic hormone; ACTH: Adrenocorticotropic hormone

Parameter	Patient value	Normal range
Glucose	91 mg/dL	60-100 mg/dL
Blood urea nitrogen	14.7 mg/dL	5-18 mg/dL
Creatinine	0.32 mg/dL	0.4-0.7 mg/dL
Sodium	131 mmol/L	138-154 mmol/L
Potassium	4.4 mmol/L	3.4-4.7 mmol/L
Calcium	6.9 mg/dL	8.8-10.8 mg/dL
Phosphorus	11.4 mg/dL	3.2-5.8 mg/dL
Magnesium	2.2 mg/dL	1.5-2.3 mg/dL
Albumin	4 mg/dL	3.8-5.4 mg/dL
PTH	< 1.20 pg/ml	1-13 pg/mL
Vitamin D	73 nmol/L	25-110 nmol/L
Cortisol	8.2 mcg/dL	5-22 mcg/dL
ACTH	14.1pg/mL	0-49 mcg/dL

The patient was evaluated by an endocrinologist who suspected an autoimmune polyglandular syndrome type I (APS1) as a possible diagnosis given the presence of chronic mucocutaneous candidiasis (CMC) and primary hypoparathyroidism. Further complementary tests revealed no data on adrenal insufficiency or other autoimmune endocrine diseases. However, the presence of anti-insulin, anti-thyroglobulin, and anti-microsomal antibodies was confirmed. An electroencephalogram (EEG) was obtained and reported as normal; therefore, her seizure was attributed to her calcium imbalance and no anticonvulsant treatment was given. Candida infection was confirmed by culture, and she was seen by an infectious disease specialist who prescribed a 12-week course of fluconazole.

**Figure 2:**
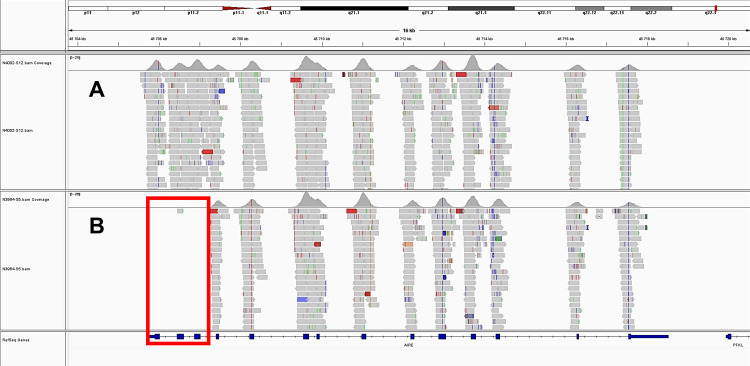
Integrative genomics viewer image of AIRE analysis by next-generation sequencing. (A) Normal sequencing of *AIRE* gene; (B) Patient sequencing where a gross homozygous deletion of the genomic region encompassing exons 1-3 of the *AIRE* gene is shown. The 5' end of this event is unknown as it extends beyond the evaluated region and, therefore, may encompass additional genes. The 3' boundary is likely confined to intron 3 of the *AIRE* gene.

A next-generation sequencing (NGS) analysis was performed by using Clinical Exome Solutionv2 and SophiaDDMTM Platform (Sophia Genetics SA, Saint Sulpice, Switzerland) in a Miseq NGS device (Illumina Inc, San Diego, California, United States). A gross homozygous deletion of the genomic region encompassing exons 1-3 of the *AIRE* gene, which includes the initiator codon, was detected (Figure 2). This alteration included the first 463 nucleotides of the *AIRE* gene coding sequence, resulting in the loss of a caspase recruitment domain (CARD domain), a homogenously staining region (HSR) domain, and a nuclear localization signals (NLS) domain. These changes are expected to result in an altered or absent protein product. After a literature review, this variant was defined as a pathogenic variant based on PVS1, PM2, and PP4 American College of Medical Genetics and Genomics (ACMG) criteria [4,5].

After normalization of electrolytic imbalances, the patient's treatment transitioned to oral calcium carbonate (1500 mg/d) with an adjusted dose of parathormone analog (1 mcg/d). She was successfully discharged with this treatment after a few weeks with normalization of electrolytes and absence of symptoms. During her follow-up appointments, significant improvement in her mucocutaneous candidiasis lesions has been seen and to this moment no other symptoms or laboratory abnormalities have been found. 

## Discussion

We described a patient with clinical criteria for APS1 and a novel homozygous* AIRE* gene mutation. She was diagnosed at a younger age when compared to other case reports in the literature [6-9], with de duad of chronic mucocutaneous candidiasis and symptomatic hypoparathyroidism, which are also the two most commonly reported manifestations [1,3]. At this point, Addison´s disease was absent but could appear later in the course of the disease, as has been described usually in older patients [3].

Among other clinical manifestations, our patient presented with malnutrition possibly associated with chronic diarrhea and malabsorption, which were present months before the diagnosis was made. She also had a dental problems history with multiple consultations due to dental cavities. Ferre et al. found that in an American cohort only 20% of the patients developed at least two of the three classic manifestations, so they proposed expanded diagnostic criteria, in which they included urticarial eruption, enamel hypoplasia, and intestinal malabsorption [6]. This alternative criterion could have helped to make an earlier diagnosis in our patient. 

No other endocrine manifestations such as thyroid diseases or diabetes mellitus were detected in our patient in the subsequent evaluations but, given the presence of autoimmune antibodies, the future risk must be considered in further evaluations. We found no other minor findings such as ophthalmologic, renal, or dermatologic alterations. However, according to what has been described in the course of this disease, close monitoring and assessment must be offered [1].

APS1 is caused by mutations in the *AIRE* gene, located in chromosome 21. It is composed of 14 exons and 545 amino acids and plays a critical role in the selection of non-autoreactive T cells [3]. There are multiple mutations described in the literature, such as the R257X in Eastern Europe, the Y855 in Iranian Jews, and the R139X in Sardinian patients [3]. However, there is no epidemiologic or genetic information in the Hispanic population and there are no published case reports of this disease in Latin America.

To our knowledge, the found mutation has not been published before and has not been previously associated with this disease. It is not described in ClinVar, Human Gene Mutation Database (HGMD®), Leiden Open (source) Variation Database (LOVD), or other databases. However, it should be defined as a pathogenic mutation based on the ACMG criteria; it is a null variant (PVS1 criteria), absent from control databases such as Genome Aggregation Database (gnomAD) and 1000 Genomes Project (PM2 criteria), and patient’s phenotype is highly specific for the disease (PP4 criteria) [4,5,10]. The consanguinity, in this case, could be relevant for these findings.

As a part of her treatment, our patient is currently receiving a PTH analog and calcium and completed a course of antifungal. This is according to the current management of APS1, which is directed towards the replacement of the hormones that are deficient [7]. She will continue follow-up with an endocrinologist given the possibility of other autoimmune manifestations and complications.

## Conclusions

Autoimmune polyglandular syndrome type I is a rare condition caused by a mutation of the *AIRE* gene that has characteristic clinical findings. In our patient, two out of three diagnostic criteria and other classic symptoms were present and thus this diagnosis was suspected. Genetic testing confirmed a homozygous deletion of the first 463 nucleotides of the *AIRE* gene, expected to result in an altered or absent protein product and defined as a pathogenic variant based on PVS1, PM2, and PP4 ACMG criteria. 
